# TNFα drives disease-specific chemokine networks and orbital tissue remodeling in a preclinical model of thyroid eye disease

**DOI:** 10.1530/ETJ-25-0407

**Published:** 2026-08-13

**Authors:** Anke Daser, Anne Gulbins, Mareike Horstmann, Jamila Kalisch, Insa Nolte, Fahimeh Hashemi Arani, Stephan Lang, Stefan Mattheis, Nikolaos E Bechrakis, J Paul Banga, Anja Eckstein, Gina-Eva Görtz

**Affiliations:** ^1^ Orbital Diseases Science Lab (ODSL), Department of Ophthalmology, University Hospital Essen, Essen, Germany; ^2^ Department of Oto-Rhino-Laryngology, Head and Neck Surgery, University Hospital Essen, Essen, Germany

**Keywords:** Graves’ disease, thyroid eye disease, preclinical mouse model, macrophages, cytokines, autoimmune disease, tumor necrosis factor (TNFα)

## Abstract

**Background:**

Thyroid eye disease (TED) is characterized by orbital inflammation, fibroblast activation, and pathological tissue remodeling. While autoimmunity to the thyrotropin receptor (TSHR) initiates disease, the contribution of proinflammatory cytokines, such as tumor necrosis factor (TNFα), to fibroblast differentiation and extracellular matrix (ECM) remodeling remains incompletely understood.

**Methods:**

A mouse model of TED was generated by immunization with a plasmid encoding the human TSHR A-subunit. Orbital tissues were analyzed for immune cell infiltration and cytokine expression. Primary murine orbital fibroblasts (mOFs) from TSHR- and control β-Gal-immunized mice were treated with TNFα or IFNγ. The expression of cytokines, chemokines, adiponectin, TGFβ, hyaluronic acid (HA), and hyaluronan synthase 2 (HAS2) was assessed by ELISA, qPCR, and western blotting.

**Results:**

TSHR-immunized mice displayed marked orbital macrophage infiltration accompanied by elevated TNFα levels. TNFα induced a strong proinflammatory response in mOFs, with robust IL-6 and IL-8 secretion and an IL-6-dominant profile in TSHR-derived cells. TNFα also upregulated CCL2, CCL20, and CXCL10, supporting enhanced immune cell recruitment. Moreover, TNFα reduced adiponectin while increasing TGFβ in TSHR mOFs, indicating activation of profibrotic pathways. TNFα significantly increased HA production and HAS2 expression, particularly in TSHR-derived fibroblasts, demonstrating enhanced ECM synthesis under autoimmune conditions.

**Conclusion:**

These findings identify TNFα as a central regulator linking inflammation to adipogenic suppression, profibrotic signaling, and ECM remodeling in a TSHR-immunized mouse model of TED. Enhanced HA/HAS2 induction underscores a disease-specific sensitivity of orbital fibroblasts to TNFα. Targeting TNFα or its downstream IL-6/TGFβ–ECM axis may offer a promising therapeutic strategy to limit pathological tissue remodeling in TED.

## Introduction

Graves’ disease (GD) is an autoimmune thyroid disease condition, mediated by autoantibodies against the thyroid-stimulating hormone receptor (TSHR). The influence of thyroid-stimulating antibodies leads to stimulation of the thyroid gland to overproduction of thyroid hormones (1). Thyroid eye disease (TED) is the most common extrathyroidal manifestation of GD. Symptoms of TED are highly variable and depend on different factors. The pathological process within the orbit includes inflammatory infiltration, expansion of adipose tissue, and glycosaminoglycan deposition in muscle and fat tissue (2, 3). This consequently results in muscle dysfunction, proptosis, and lid retraction (4, 5, 6). In rare cases, TED can lead to blindness (7). In TED, orbital fibroblasts are sentinel pathogenic cells that respond to TSHR antibodies, inflammatory T cells, and cytokines (8). Chemokines are released in response to signals such as proinflammatory cytokines and play an important role in the recruitment of monocytes, neutrophils, and lymphocytes to the diseased orbital tissue. The migration of cells expressing the corresponding chemokine receptor occurs along a chemokine gradient, which allows the cells to move toward high local concentrations of chemokines (9). Consistent with this concept, retro-orbital tissues from TED patients demonstrate diffuse lymphocytic infiltration and lymphoid aggregates (10), underscoring the importance of chemokine-driven immune cell recruitment in orbital pathology. Other studies suggest that chemokines play an immunomodulatory role in the pathogenesis of GD, particularly in its extrathyroidal manifestation, TED. Various chemokines, such as CXCL10, CCL2, and CXCL8, and cytokines, such as tumor necrosis factor (TNFα), have been found to be elevated in the serum and orbital tissue of patients with active TED, indicating a predominantly proinflammatory role (11). In addition, circulating TSHR-expressing fibrocytes in TED patients amplify the inflammatory milieu by the secretion of proinflammatory chemokines in orbital tissue (12). The combination of the chemokines and cytokines is thought to contribute to disease progression by promoting the recruitment and activation of immune cells, including Th1 cells, monocytes, and neutrophils, into the orbital tissue. In line with this, the presence of macrophage-derived cytokines, such as IL-1β, TNFα, and IL-10, suggests ongoing macrophage activation and antigen presentation in orbital tissue (12).

The therapeutic relevance of targeting inflammatory pathways, such as the TNFα pathway, is further supported by studies in other autoimmune diseases. For example, Balato *et al.* demonstrated that treatment with adalimumab, a TNFα-blocking monoclonal antibody, resulted in substantial clinical improvement and suppression of the Th17 response in patients with moderate-to-severe psoriasis (13). Similarly, other TNFα inhibitors, such as etanercept and golimumab, have shown beneficial anti-inflammatory effects in autoimmune diseases. In TED, a pilot study by Paridaens *et al.* reported improvement in inflammatory disease activity following etanercept treatment (14), supporting the concept that TNFα-dependent signaling contributes to orbital inflammation and may represent a potential therapeutic target in TED.

Current therapeutic strategies for thyroid eye disease (TED), most notably the IGF-1R-targeting antibody teprotumumab (Tepezza), have demonstrated substantial clinical efficacy, particularly in reducing proptosis and inflammatory activity. However, treatment responses are variable, a subset of patients show limited or no improvement, and adverse effects, including hearing impairment and metabolic disturbances, have been reported (15, 16, 17). Moreover, Tepezza primarily targets the IGF-1R pathway and may not fully address the broader inflammatory network underlying TED pathogenesis. These limitations underscore the need to better characterize additional immune mechanisms driving disease progression. In this context, alternative inflammatory pathways, such as TNFα signaling, may represent complementary or independent therapeutic targets. By investigating the role of TNFα and its effects on orbital fibroblast function, our study aims to address an important gap in current therapeutic strategies and may contribute to the development of more targeted or combinatorial treatment approaches for patients who show insufficient responses to existing therapies.

Recently, we have established a preclinical model of TED by genetic immunization of BALB/c mice with plasmids encoding the A-subunit of human TSHR (18, 19, 20). Importantly, we showed in our mouse model the early infiltration of F4/80-expressing macrophages into the orbital tissue, prior to influx of inflammatory T cell populations (21). Moreover, splenic T cells from TSHR-A-subunit-primed animals undergoing TED showed an antigen-specific proliferative response to purified TSHR antigen accompanied by secretion of proinflammatory cytokines, including IFNγ, IL-10, IL-6, TNFα, and CCL20, which may play an important role in the recruitment of macrophages and orbital manifestations in TED (22). This model closely recapitulates key immunological and orbital features of human TED and therefore represents a valuable tool for investigating disease mechanisms and potential therapeutic targets.

Therefore, the aim of this study was to investigate the role of TNFα in mediating disease-specific activation of orbital fibroblasts in a TSHR-immunized mouse model of TED. We hypothesized that TNFα induces a distinct proinflammatory and profibrotic response in fibroblasts derived from diseased animals, thereby contributing to immune cell recruitment and tissue remodeling beyond general inflammatory activation. Furthermore, this study could provide additional insights into TNFα-mediated signaling pathways in TED and may help support further research into anti-TNFα treatment strategies.

## Methods

### Mice

Female BALB/c mice of age 6–8 weeks were purchased from Envigo, the Netherlands, and housed in a specific pathogen-free environment in animal care facilities at University Hospital Essen. Animal procedures were reviewed and approved by State Agency for Consumer Protection and Food of North Rhine-Westphalia (LAVE), Germany. Animals were housed for one week to acclimate and were then immunized with 50 μg (1 mg/mL) TSHR A-subunit plasmid or control β-Gal l plasmid by intramuscular injection followed by electroporation into each biceps femoris muscle. Immunizations were performed four times at three-week intervals. A total number of 31 animals were immunized, 14 with TSHR A-subunit plasmid and 17 animals with control β-Gal plasmid. Mice were sacrificed 6 weeks after the last immunization, as described in our earlier studies (18, 19, 20, 22, 23).

### Culture of mOFs

One orbit of each mouse was formalin-fixed and used for immunohistochemistry, as described (18, 19, 20, 23). The other orbit was excised under aseptic conditions, and the retrobulbar tissue was dissected by careful removal of the Harderian gland for culture of murine orbital fibroblasts (mOFs) (24). Briefly, the tissue was cultured with the standard medium: DMEM (Invitrogen, USA) supplemented with 10% fetal calf serum (Sigma-Aldrich, USA), 10 mM HEPES (pH 7,4), 2 mM-glutamine, 1 mM sodium pyruvate, 100 U/mL penicillin, and 100 μg/mL streptomycin (Pan Biotech, Germany). Primary mOF cultures were successfully established from a subset of immunized animals undergoing GD/TED in the group. Due to variability in tissue yield and cell outgrowth, stable fibroblast cultures were generated from 8 β-Gal control mice and 8 TSHR-immunized mice. After 3–4 weeks, the orbital tissue and non-adherent cells were removed by washing with PBS. Once cells grew in adherent monolayers, they were transferred to a 75-mL flask, serially passaged and maintained in the medium described above. All experiments were performed with mOFs between passages 2 and 5 (24).

### Immunohistochemistry

Consecutive 1 μm sections of the middle area of orbital tissues were stained with antibodies for F4/80 (1:100, Bio-Rad, UK), TNFα (1:100, Elabscience, USA), and CD3 (1:25, Agilent Dako, USA) using an HRP-conjugated polymer system in accordance with the manufacturer’s manual (Zytomed Systems GmbH, Germany). F4/80- and CD3-positive cells were counted in muscle, peri-neural connective tissue and fat tissue. Images were generated using an Olympus BX51 microscope.

### LEGENDplex and Bio-Plex analysis

Independent lines of mOF derived from TSHR-immunized mice and β-Gal control mice (*n* = 8 lines from each group) were seeded at a density of 1 × 10^4^ cells per well on coverslips in 96-well plates. They were grown in standard culture medium, starved for 24 h with standard medium containing 1% FCS and stimulated with TNFα (10 ng/mL, R&D, USA) and INFγ (1000 U/mL, Peprotech, USA) for 48 h. Supernatants were analyzed via LEGENDplex (BioLegend, USA) assay and Bio-Plex Pro Assay (Bio-Rad, UK; Lot# 50450099) according to the manufacturer’s instructions.

### Real-time quantitative PCR

mOFs (*n* = 8 independent cell lines per group, derived from individual mice) were seeded in 6-well plates at 3 × 10^5^ cells per well, cultured in standard medium, serum-starved for 24 h (1% FCS), and stimulated with TNFα (10 ng/mL; R&D Systems) for 48 h. Total RNA was isolated using the guanidinium isothiocyanate method (32), and 1 μg RNA was reverse-transcribed with oligo(dT) primers. qRT-PCR was performed using Takyon No ROX SYBR MasterMix (Eurogentec, Belgium) on an Applied Biosystems, USA, StepOnePlus system. Reactions (25 μL) contained 0.5 μL cDNA and 10 pmol of each primer. Gene expression was normalized to β-actin and is shown as relative mRNA levels. All assays were run in technical duplicates using RNA from three independent biological replicates. Primer sequences are listed in Table 1.

**Table 1:** Real-time PCR primer sequences.

Genes	Primer Sequences, 5’→ 3’	
	Forward	Reverse
CCL2	CAC​TCA​CCT​GCT​GCT​ACT​CA	GCT​TGG​TGA​CAA​AAA​CTA​CAG​C
*CCL5*	ACC​ATA​TGG​CTC​GGA​CAC​CA	TCT​CTG​GGT​TGG​CAC​ACA​CTT
*CCL20*	AGC​TTC​ATC​GGC​CAT​CTG​TC	CAG​GCA​GAA​GCA​AGC​AAC​TAC
*CXCL1*	GGC​TTG​CCT​TGA​CCC​TGA​AG	CGT​TCA​CCA​GAC​AGG​TGC​CA
*CXCL10*	CTG​CTG​GGT​CTG​AGT​GGG​AC	ATC​TCA​ACA​CGT​GGG​CAG​GA
Adiponectin (AdipoQ)	CTG​CAA​CAT​TCC​GGG​ACT​CT	TGA​AGA​GAA​CGG​CCT​TGT​CC
*TGFß1*	GAC​CCC​CAC​TGA​TAC​GCC​TG	TGG​GGC​TGA​TCC​CGT​TGA​TTT
*HAS2*	AAA​GGG​CCT​GCC​AGT​CTT​AT	CCT​GTT​GGT​AAG​GTG​CCT​GT

### Migration assay

mOFs (*n* = 4 independent cell lines per group, derived from individual mice) were seeded at a density of 1 × 10^5^ cells per well in 24-well plates and serum-starved for 24 h (DMEM supplemented with 1% FCS), followed by stimulation with 10 ng/mL TNFα for 72 h at 37°C. PMA-stimulated (phorbol-12-myristate-13-acetate, 10 ng/mL, 48 h) and LPS-stimulated (lipopolysaccharide, 100 ng/mL, 72 h) murine monocyte macrophages (J774A.1, DSMZ, Leibniz Institute, Braunschweig, Germany) were seeded into transwell inserts (PET membrane, 8 μm pore size; Sarstedt AG & Co. KG, Germany) in DMEM without FCS and placed into wells containing mOFs derived from TSHR- or ß-Gal-immunized mice. After 24 h incubation at 37°C, non-migrated macrophages on the upper side of the membrane were carefully removed. Inserts were then fixed and Giemsa-stained. Membranes were mounted on glass slides, and macrophages that had migrated to the lower side of the membrane were counted microscopically using a 10× objective (Olympus BX51, Upright). Migrated macrophages were normalized to the total number of macrophages initially seeded into the insert and expressed as percentage of migrated cells.

### Multiplex cytokine analysis

TNFα and IFNγ levels were measured in orbital connective tissue using a Luminex Discovery Assay (mouse premixed multi-analyte Kit, R&D Systems, Cat# LXSAMSM), following the manufacturer’s protocol. Measurements were acquired on a Bio-Plex System (Bio-Rad Laboratories, USA). Cytokine concentrations were normalized to the total protein content of each sample.

### Hyaluronan ELISA

Cultures of individual orbital tissue-derived mOFs were seeded at a density of 1 × 10^4^ cells per well on 96-well dishes and grown in standard culture medium. After one day, the medium was exchanged with a low-serum medium (1%). The cells were pretreated for 1 h at 37°C with hyaluronidase (1U/mL; Sigma-Aldrich, Merck, Germany) and stimulated with TNFα (10 ng/mL) for 48 h. Hyaluronan levels in the supernatants were measured using an ELISA kit (R&D Systems, Germany) according to the manufacturer’s instructions. Samples were diluted 1:50 before analysis.

### Western blot

mOFs were lysed in 50 μL extraction buffer (300 mM NaCl, 10 mM Tris/HCl pH 7.9, 1 mM EDTA, 0.1% Nonidet P-40, and protease inhibitors) for 20 min on ice. Protein concentrations were measured using a commercial assay (Bio-Rad). For western blotting, 30 μg protein per lane was separated on 12% SDS-PAGE and transferred to nitrocellulose membranes. Membranes were blocked in 5% skimmed milk/TBST for 1 h and incubated overnight at 4°C with primary antibodies against adiponectin (1:1,000, CST #2789) and TGFβ (1:1,000, Abcam, UK (ab215715)). After TBST washes, membranes were incubated with HRP-conjugated anti-rabbit IgG (1:2,000) for 1 h at room temperature, and signals were detected using an ECL system. Membranes were reprobed with antibodies against GAPDH (CST #2118) or β-actin (CST #4970) as loading controls. Band intensities were quantified using ImageJ, and adiponectin and TGFβ levels were normalized to GAPDH or β-actin.

### Statistical analysis

Statistical analyses were performed using GraphPad (Prism 7). The level of significance was set at *P* < 0.05. Statistical analyses were carried out with two-tailed Student’s t-tests or two-way ANOVA. Data are presented as arithmetic mean ± SEM. *P* values are marked with asterisks representing *P < 0.05; **P < 0.01; ***P < 0.001; and ****P < 0.0001.

To assess the proportional contribution of IL-6 and IL-8 to the TNFα-induced inflammatory response, an exploratory cytokine index was calculated based on log-transformed fold changes. For each cytokine, the fold induction between stimulated and unstimulated conditions was transformed to log_2_ scale. The index was defined as follows:

Cytokine index = Δlog_2_(IL-6) – Δlog_2_(IL-8).

Positive index values indicate a relatively stronger IL-6 induction, whereas negative values indicate an IL-8-dominant response. In contrast to conventional statistical analyses, which evaluate IL-6 and IL-8 independently, this index was designed to assess the relative balance between both cytokines within the same inflammatory response. The cytokine index was therefore used as an exploratory analytical tool to visualize qualitative shifts in cytokine polarization rather than as a validated biomarker or stand-alone quantitative parameter.

## Results

### TSHR immunization induces orbital inflammation and upregulation of TNFα in a mouse model of TED

**Figure 1:**
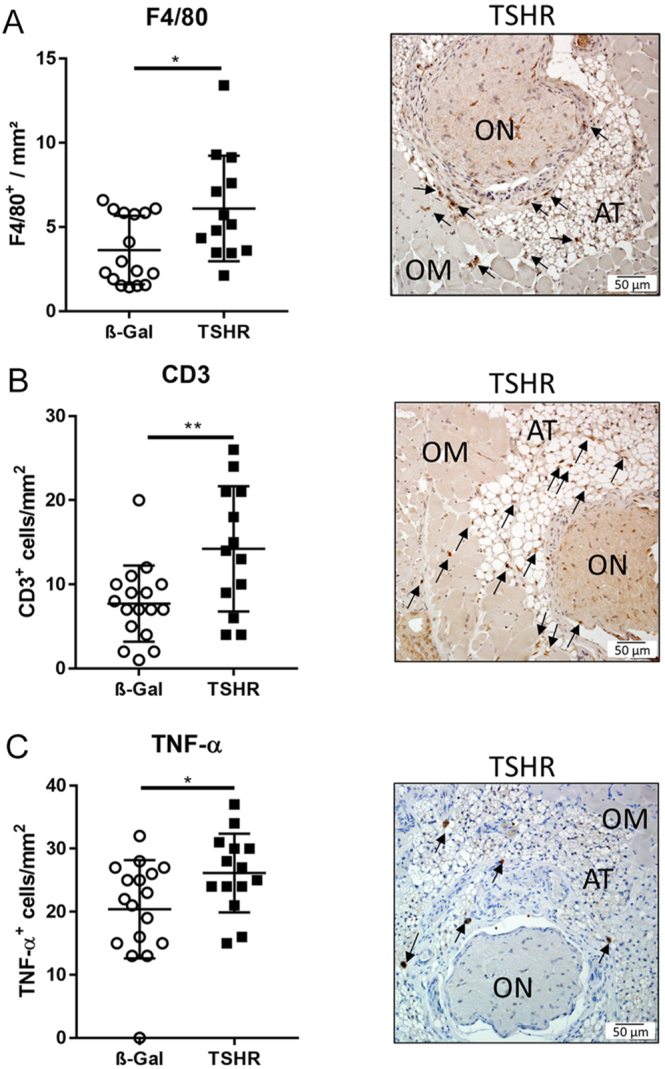
Histological analyses of orbital tissues. Orbital sections from TSHR-immunized mice (*n* = 17) and β-Gal controls (*n* = 14) were stained for (A) F4/80-positive macrophages, (B) CD3-positive T cells, and (C) TNFα-positive cells. Representative images from TSHR-immunized mice are shown. The arrows indicate positively stained cells. Positive cells were quantified per mm^2^ in defined orbital regions, including ocular muscle (OM), connective tissue, adipose tissue (AT), and around the optic nerve (ON). Images were acquired using an Olympus BX51 microscope (magnification ×400). Data are presented as mean ± SEM. Statistical analysis was performed using Student’s *t*-test (*P < 0.05, **P < 0.01).

**Figure 2:**
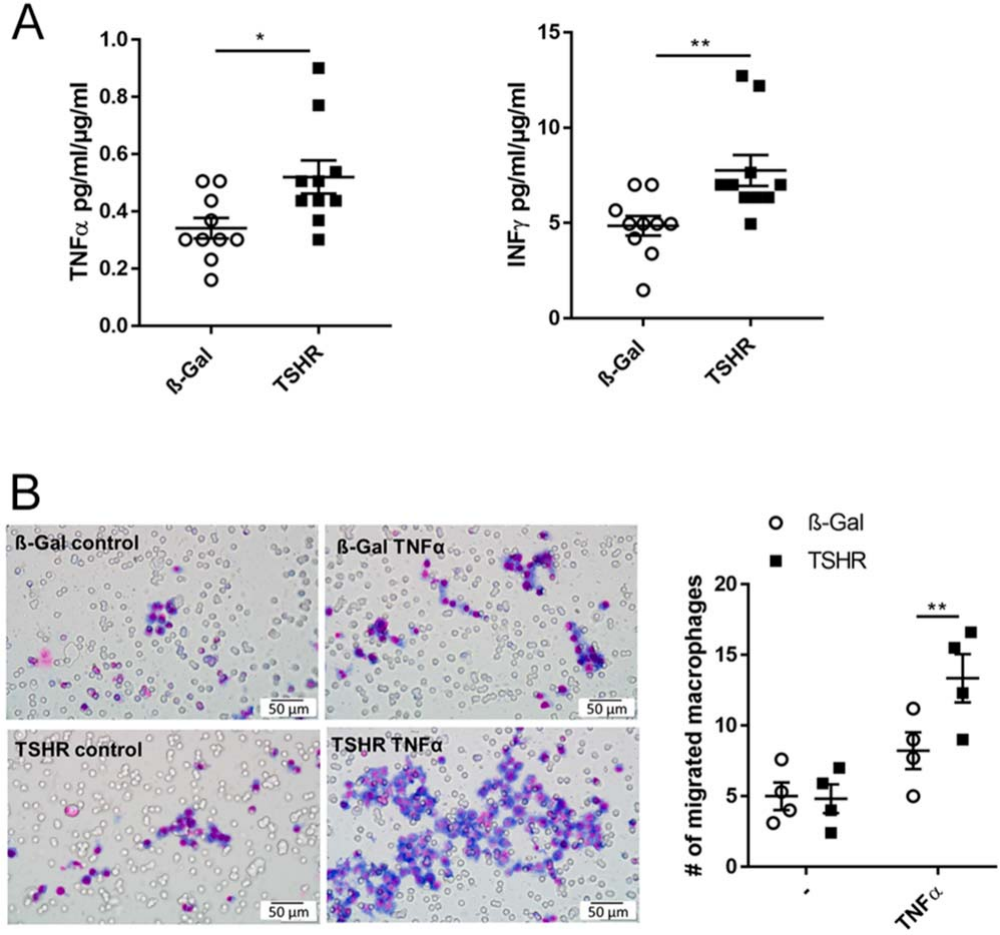
Effect of TNFα on the migration of macrophages. (A) TNFα and IFNγ protein levels in orbital connective tissue from TSHR-immunized mice (*n* = 8) and β-Gal control (*n* = 8) and were quantified using a magnetic Luminex assay and normalized to total protein content. Each data point represents one individual mouse. (B) Macrophage migration assay: mOFs derived from TSHR-immunized and β-Gal control mice (*n* = 4 independent cell lines per group) were stimulated with TNFα (10 ng/mL) or left unstimulated. Migration of macrophages toward mOFs was quantified after 24 h using a transwell insert system. Non-migrated cells on the upper side of the membrane were removed, and migrated macrophages on the lower side were fixed, Giemsa-stained, and quantified microscopically. Representative images of Giemsa-stained membranes are shown. Each data point represents one independent mOF line. Images were acquired using an Olympus BX51 microscope. Data are shown as mean ± SEM. Statistical analysis was performed using two-way ANOVA (**P < 0.01).

Orbital tissues from mice were histologically examined to assess immune cell infiltration and cytokine expression. Serial sections of the central orbital region were immunohistochemically stained for F4/80 (macrophage marker), CD3 (T cell marker), and TNFα (proinflammatory cytokine), as previously described (18, 21, 25). Compared to control animals, TSHR-immunized mice showed a significantly increased infiltration of both macrophages and T cells in the retro-orbital tissue (Fig. 1A and B). In addition, a higher number of TNFα-positive cells were detected in the orbital fat tissue surrounding the optic nerve of TSHR-immunized mice undergoing experimental TED (Fig. 1C). To further quantify cytokine expression in the orbital connective tissue, a multiplex analysis was performed. This confirmed marked upregulation of TNFα and IFNγ in the orbital tissue of TSHR-immunized mice compared to controls (Fig. 2A). The concurrent elevation of TNFα and IFNγ, two hallmark Th1 cytokines, suggests activation of a Th1-dominated inflammatory response in the orbit. While TNFα is known to promote immune cell recruitment and tissue remodeling, IFNγ may enhance these effects by further activating local fibroblasts and sustaining the proinflammatory environment. These findings confirm the successful induction of an inflammatory orbital phenotype through TSHR immunization, supporting the relevance of this model for studying the pathogenesis of TED.

### TNFα-stimulated mOFs promote macrophage migration in a TED-associated context

Based on the observed increase in TNFα-positive cells within the orbital tissue of TSHR-immunized mice, we next investigated the functional impact of elevated TNFα levels on mOFs. In particular, we aimed to determine whether TNFα stimulation of mOFs influences macrophage migration, a key feature of the inflammatory response in TED.

To this end, mOFs from both β-Gal control and TSHR-immunized mice were stimulated with TNFα, and their ability to attract macrophages was assessed in a migration assay. TNFα-stimulated mOFs promoted increased macrophage migration in both groups compared to non-stimulated cells (Fig. 2B). Notably, mOFs from TSHR-immunized mice induced a significantly higher migration than those from control mice, indicating a disease-associated amplification of TNFα-mediated immune cell recruitment.

### Cytokine and chemokine expression is increased upon TNFα stimulation

**Figure 3:**
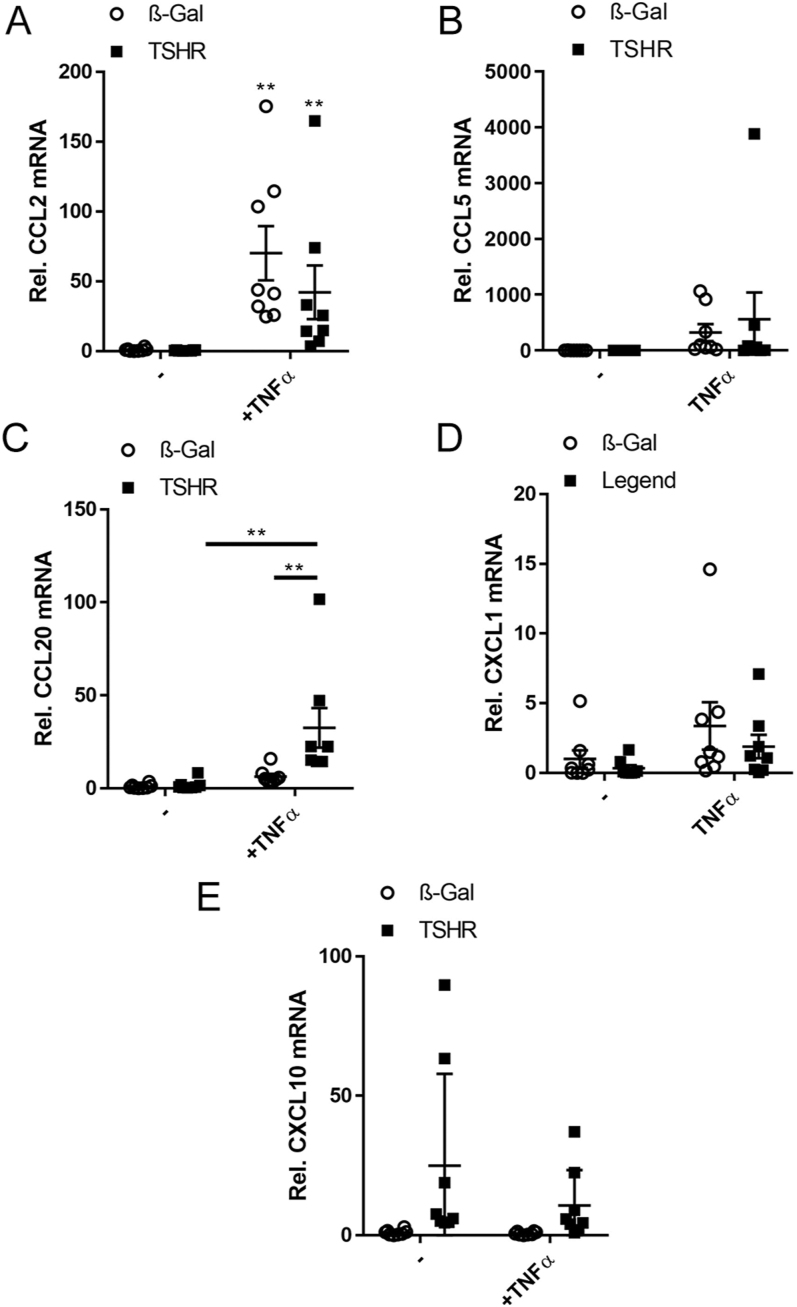
TNFα-induced chemokine mRNA expression in murine orbital fibroblasts (mOFs). (A, B, C, D, E) mOFs derived from TSHR-immunized mice and β-Gal controls (*n* = 8 independent cell lines per group) were stimulated with TNFα (10 ng/mL) for 24 h. mRNA expression levels of indicated chemokines were quantified by RT-PCR. Each data point represents one independent biological replicate (individual mOF line), measured in technical duplicates. Data are presented as mean ± SEM. Statistical analysis was performed using two-way ANOVA as appropriate (*P < 0.05, **P < 0.01, ***P < 0.001, ****P < 0.0001).

**Figure 4:**
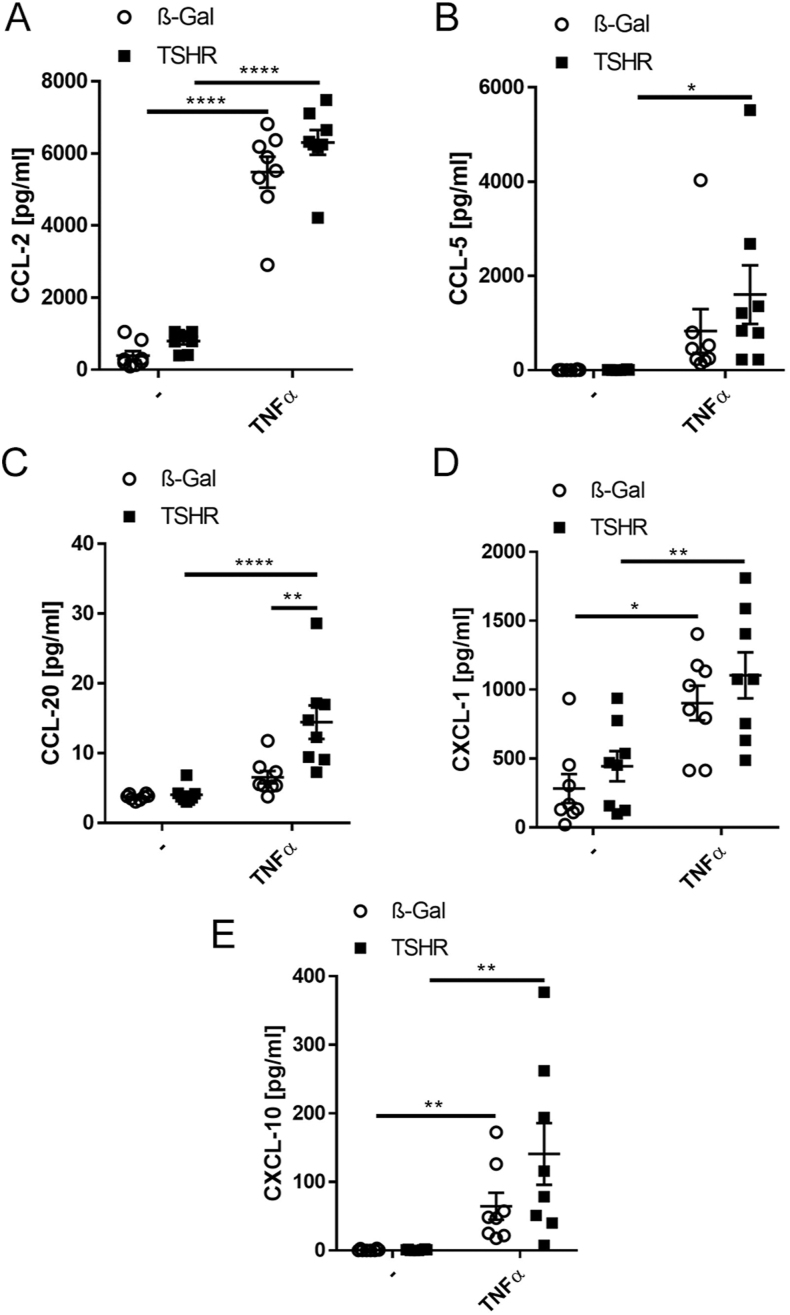
TNFα-induced chemokine protein secretion in murine orbital fibroblasts (mOFs). (A, B, C, D, E) Supernatants from TNFα-stimulated mOFs (10 ng/mL, 24 h) derived from TSHR-immunized and β-Gal control mice (*n* = 8 independent cell lines per group) were analyzed using a LEGENDplex assay. Each data point represents one independent mOF line. Data are shown as mean ± SEM. Statistical analysis was performed using two-way ANOVA (*P < 0.05, **P < 0.01, ***P < 0.001, ****P < 0.0001).

TNFα-stimulated mOFs were further analyzed with RT-PCR and LEGENDplex assay to identify relevant cytokines that play a role in the immune response of TED. The inflammatory panel of cytokines showed no relevant statistically significant correlation (data not shown). In the proinflammatory panel, TNFα-stimulated mOFs had an increased response in numerous cytokines (Figs 3 and 4). TNFα-stimulated mOFs derived from both TSHR and β-Gal control mice showed increased expression of CCL2 in comparison with unstimulated cells. CCL5 was found increased in the cell lysates and supernatant of stimulated mOFs derived from TSHR mice. Besides increased expression in stimulated TSHR mOFs in comparison with unstimulated cells, CCL20 showed furthermore a statistically significant increase in TNFα-stimulated TSHR mOFs compared to TNFα-stimulated control mOFs. Moreover, the chemokines CXCL1 and CXCL10 were also increased upon TNFα stimulation.

### TNFα links inflammatory signaling with extracellular matrix remodeling and profibrotic responses in orbital fibroblasts from TSHR-immunized mice

Given the central role of TNFα in the inflammatory microenvironment of TED, its effects on adipogenic, profibrotic, and proinflammatory pathways were analyzed in mOFs derived from TSHR- and control β-Gal-immunized mice.

**Figure 5:**
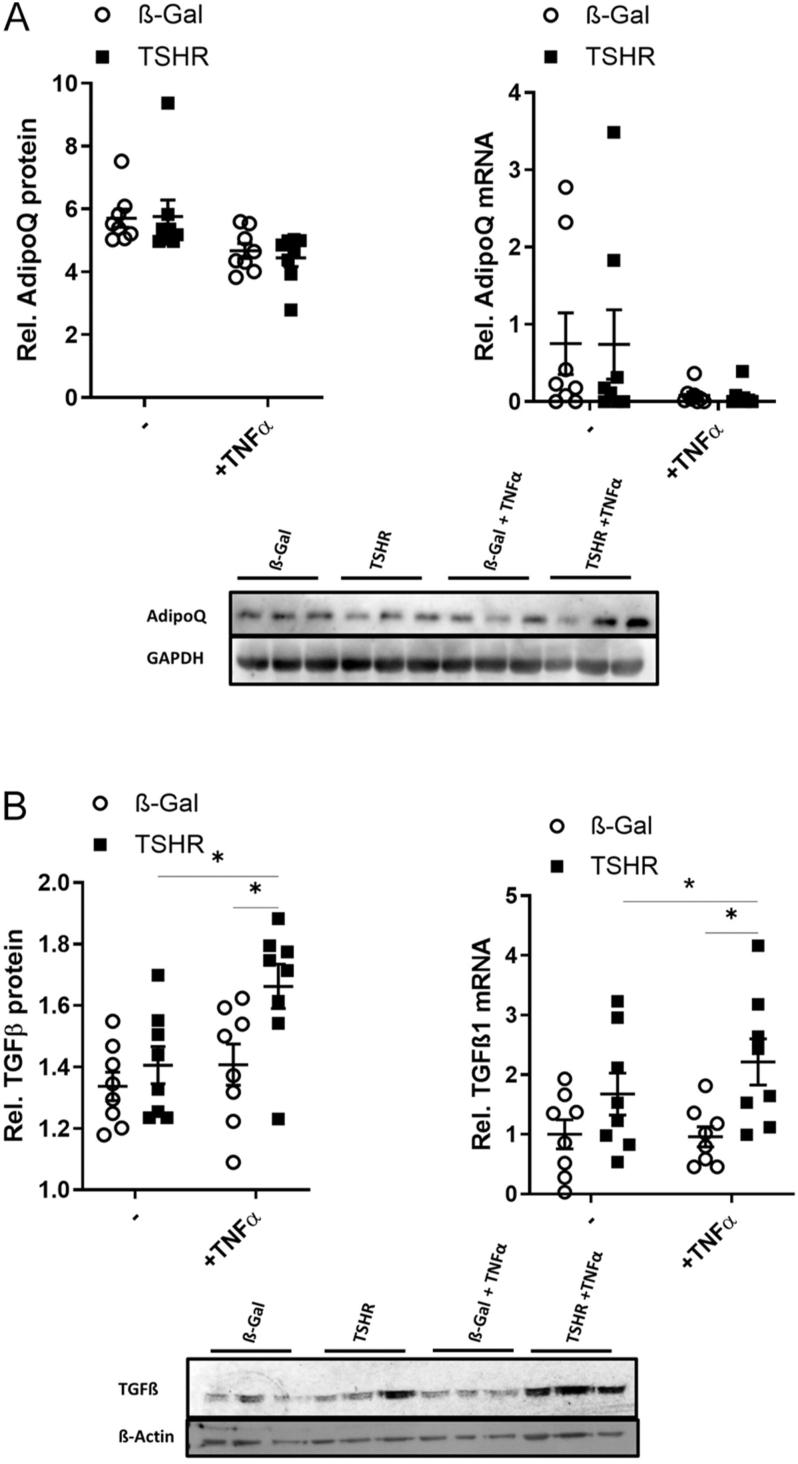
TNFα differentially regulates adiponectin and TGFβ expression in murine orbital fibroblasts (mOFs) from TSHR-immunized versus control mice. (A) Adiponectin protein and mRNA expression levels in mOFs derived from TSHR-immunized and β-Gal control mice (*n* = 8 independent cell lines per group) were analyzed by western blot and RT-PCR following 24 h stimulation with TNFα (10 ng/mL). (B) TGFβ protein and mRNA expression under basal and TNFα-stimulated conditions. GAPDH or β-actin served as loading controls for western blot analyses, while the housekeeping gene β-actin was used for normalization of RT-PCR data. Band intensities and relative mRNA expression levels were quantified and normalized. Each data point represents one independent mOF line. Representative western blots are shown. Data are presented as mean ± SEM. Statistical analysis was performed using two-way ANOVA (*P < 0.05, **P < 0.01).

Western blot analyses revealed that TNFα stimulation resulted in a moderate reduction in adiponectin protein expression in mOFs from both groups, although this decrease did not reach statistical significance (Fig. 5A). This trend indicates that TNFα exerts a mild inhibitory effect on adipogenic differentiation. In contrast, TNFα markedly upregulated TGFβ expression in TSHR mOFs, whereas no increase was observed in control mOFs (Fig. 5B). Basal TGFβ levels did not differ significantly between the groups, suggesting that the profibrotic response is specifically induced by TNFα stimulation and could be more pronounced in orbital fibroblasts derived from animals undergoing TED.

**Figure 6:**
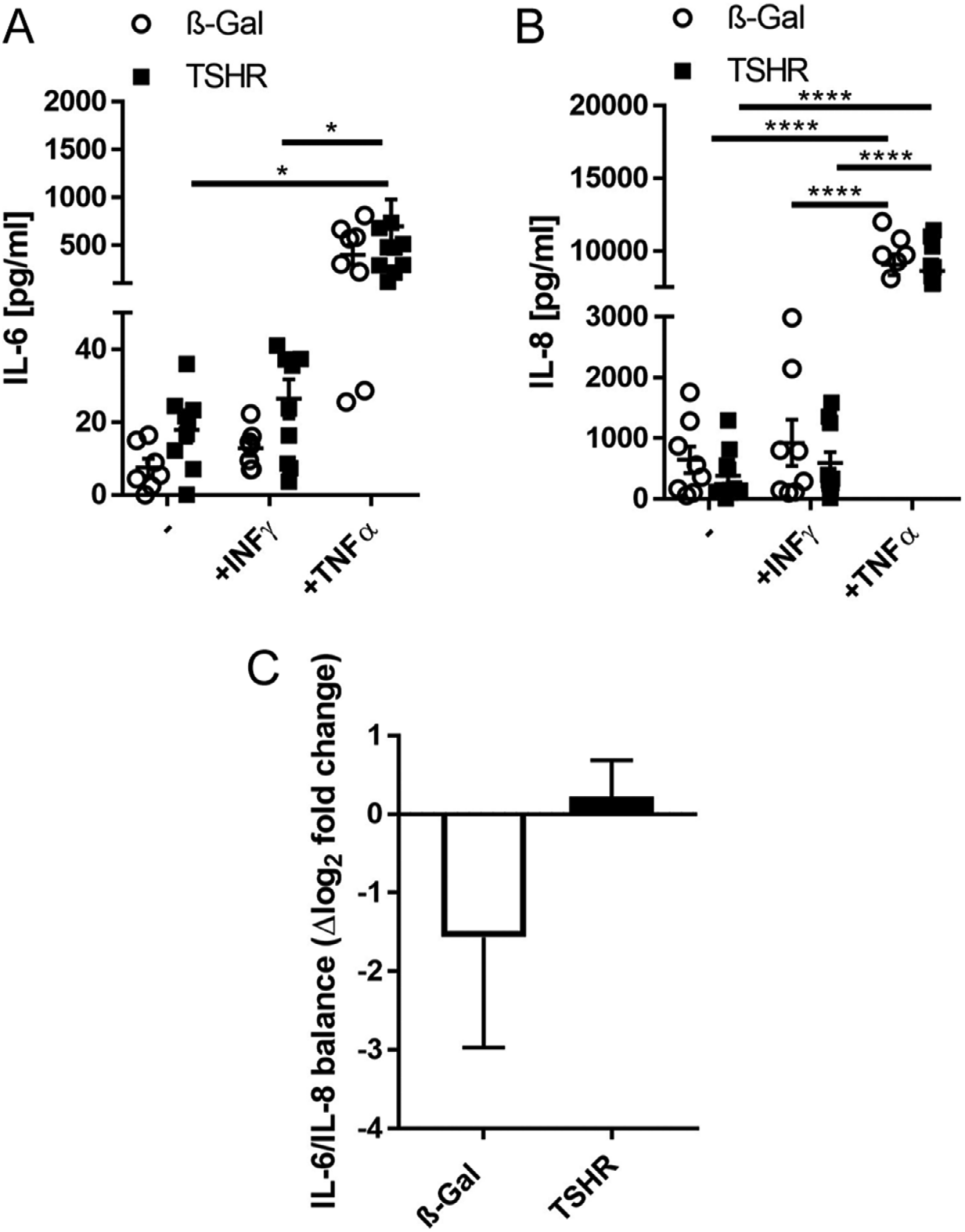
TNFα-induced proinflammatory cytokine response in orbital fibroblasts. (A, B) IL-6 and IL-8 concentrations in supernatants of mOFs derived from TSHR-immunized and β-Gal control mice (*n* = 8 independent cell lines per group) were measured by ELISA after 24 h stimulation with TNFα or IFNγ. (C) Combined index Δlog_2_(IL-6) – Δlog_2_(IL-8) representing the relative shift in the TNFα-induced cytokine response. Positive values indicate an IL-6-dominant response, whereas negative values reflect an IL-8-dominant response. The bars represent mean ± SEM (*P < 0.05, **P < 0.01, ***P < 0.001, ****P < 0.00001).

To further characterize the inflammatory activation of mOFs, analyses for IL-6 and IL-8 were evaluated by ELISA. TNFα induced a robust proinflammatory response, leading to significantly increased secretion of both cytokines (Fig. 6A and B). The induction of IL-6 was significantly stronger in TSHR-derived fibroblasts compared to β-Gal controls. In contrast, stimulation with IFNγ did not significantly alter IL-6 or IL-8 secretion, suggesting a more prominent role for TNFα in driving the inflammatory response in this experimental setting.

To integrate these findings, a combined cytokine index (Δlog_2_(IL-6) – Δlog_2_(IL-8)) was calculated to represent the TNFα-dependent shift between IL-6 and IL-8 induction. This index reflects the proportional shift between both cytokines, independent of absolute secretion levels. Positive values indicate a relative IL-6 dominance, whereas negative values reflect an IL-8-dominated response. This index was significantly higher in mOFs from TSHR-immunized mice than from β-Gal controls (Fig. 6C), suggesting a TNFα-mediated shift toward an IL-6 dominated, profibrotic cytokine profile.

**Figure 7:**
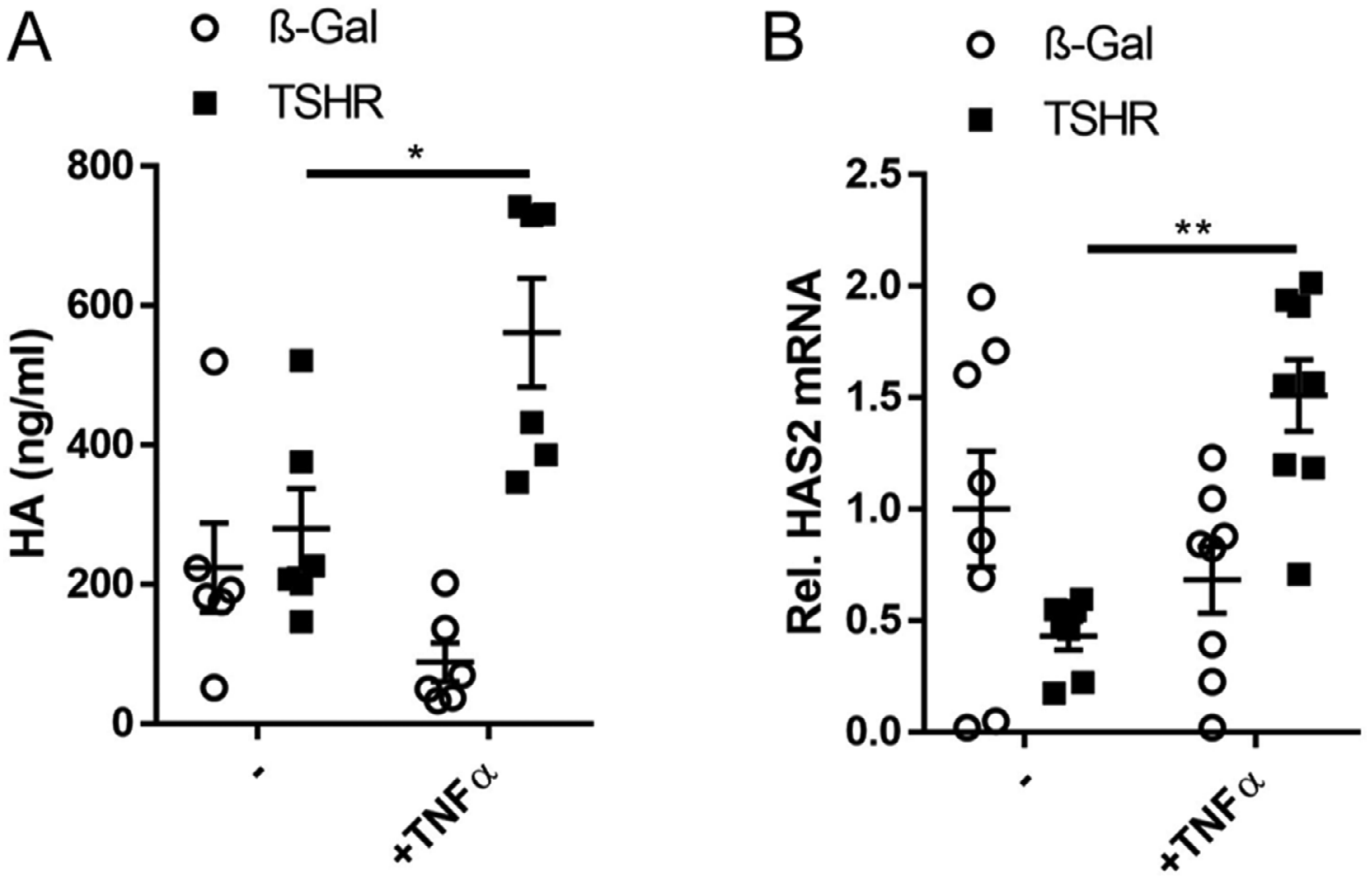
TNFα promotes hyaluronic acid (HA) production and HAS2 expression in orbital fibroblasts from TSHR-immunized mice. (A) HA concentrations in supernatants of mOFs derived from TSHR-immunized and β-Gal control mice (*n* = 8 independent cell lines per group) were quantified by ELISA after 48 h stimulation with TNFα (10 ng/mL). (B) HAS2 mRNA expression from TSHR-immunized and β-Gal control mice (*n* = 8 independent cell lines per group) was analyzed by RT-PCR under basal and TNFα-stimulated conditions. Each data point represents one independent biological replicate (individual mOF line). Data are presented as mean ± SEM. Statistical analysis was performed using two-way ANOVA (*P < 0.05, **P < 0.01).

Given the characteristic glycosaminoglycan accumulation in TED, we next assessed whether TNFα affects hyaluronic acid (HA) production in orbital fibroblasts. Consistent with its profibrotic and proinflammatory effects, TNFα stimulation significantly increased HA concentrations in mOF supernatants, with a more pronounced induction in cells from TSHR-immunized mice than from β-Gal controls (Fig. 7A). This was accompanied by elevated expression of the HA-synthesizing enzyme hyaluronan synthase 2 (HAS2) (Fig. 7B), indicating activation of the HA synthetic pathway.

Together with the upregulation of TGFβ and IL-6, these findings demonstrate that TNFα not only promotes inflammatory and profibrotic signaling but also drives extracellular matrix (ECM) remodeling in a disease-specific manner in orbital fibroblasts.

## Discussion

This study provides novel insights into the inflammatory mechanisms driving TED associated with GD in a preclinical mouse model, emphasizing the pivotal role of TNFα and its downstream chemokine network in modulating fibroblast function, immune cell recruitment, and tissue remodeling. An important aspect of our study is the identification of a disease-specific TNFα response signature in orbital fibroblasts derived from mice undergoing experimental TED, linking inflammatory signaling directly to altered fibroblast phenotype and ECM remodeling. While TNFα is a well-known proinflammatory cytokine, our data suggest that the autoimmune context modifies fibroblast responsiveness, resulting in enhanced chemokine production, macrophage recruitment, and profibrotic signaling. Importantly, these findings extend previous observations in human TED by providing mechanistic evidence that autoimmune priming sensitizes orbital fibroblasts to TNFα-driven pathogenic activation.

Our findings are consistent with prior studies demonstrating elevated TNFα levels in orbital tissues of TED patients (12, 26, 27). TNFα induces several CC and CXC chemokines relevant for leukocyte recruitment and chronic inflammation, including CCL2, CCL5, CCL20, CXCL1, and CXCL10 (9, 11, 28, 29, 30). CCL2 (MCP-1) is a well-characterized chemokine that plays a central role in recruiting monocytes and macrophages to sites of tissue inflammation. In the context of TED, increased CCL2 levels have been reported in the serum, orbital tissues, and cultured orbital fibroblasts of affected patients (27, 29), implicating this chemokine in disease pathogenesis. In our TSHR-immunized mouse model, TNFα robustly induced CCL2 expression in orbital fibroblasts, confirming its responsiveness to proinflammatory stimuli. However, this induction was not disease-specific, as control fibroblasts also responded to TNFα stimulation. This suggests a general inflammatory response rather than a TED-specific fibroblast phenotype.

In contrast, CCL20 expression was significantly enhanced in TSHR-derived fibroblasts, indicating disease-specific activation of the CCL20/CCR6 axis. CCL20 is a key chemoattractant for CCR6^+^ Th17 cells and has previously been implicated in TED pathogenesis and other autoimmune conditions (27, 29, 31, 32, 33). The pronounced induction observed in our model supports the concept that orbital fibroblasts may actively contribute to Th17-mediated inflammation in TED. Future studies should further investigate whether increased CCL20 expression promotes recruitment of pathogenic Th17 and Th17.1 cells, which have recently been associated with severe and steroid-resistant TED through secretion of IL-17A and IFNγ (34). Importantly, our data suggest that autoimmune priming amplifies TNFα-dependent Th17-associated signaling pathways, which may contribute to persistent orbital inflammation in severe disease stages.

Similarly, TNFα significantly increased CXCL1 and CXCL10 expression. CXCL1 is a known neutrophil chemoattractant induced by IL-17A, which has been implicated in TED. CXCL10, a well-established IFNγ and TNFα-inducible chemokine, is elevated in TED and correlates with disease activity (29, 35). In contrast, CCL5 expression was not significantly altered following TNFα stimulation, which accords with previous observations in human TED orbital fibroblasts (27, 36). This finding suggests that CCL5 induction likely requires additional inflammatory signals, such as IFNγ-, IL-17A-, or CD40L-mediated co-stimulation, as previously described in chronic inflammatory settings.

Our *in vivo* studies in an experimental mouse model of TED highlight TNFα as a key regulator of orbital fibroblast activity, orchestrating adipogenic, profibrotic, and proinflammatory pathways. The *in vivo* model also demonstrates that the proinflammatory milieu in orbital tissue in animals undergoing TED alters fibroblast responsiveness to inflammatory stimuli, thereby identifying TNFα as a potential upstream driver of pathological tissue remodeling in TED. In mOFs derived from TSHR-immunized mice, TNFα stimulation attenuated adipogenic differentiation, as evidenced by reduced adiponectin expression, while simultaneously enhancing TGFβ expression, a key mediator of fibrotic remodeling. This response was not observed in fibroblasts from β-Gal-immunized control mice, indicating that the proinflammatory milieu in autoimmunity confers an increased sensitivity of orbital fibroblasts to TNFα-induced profibrotic activation. This disease-specific responsiveness is particularly relevant because it suggests that inflammatory signaling pathways in TED are not merely quantitatively increased, but qualitatively altered, which may help explain persistent inflammation and fibrosis in patients with progressive disease. These findings are consistent with earlier studies that reported an inhibitory effect of TNFα on adipogenesis. Valyasevi *et al.* (27) demonstrated that TNFα and IFNγ inhibited adipogenesis and downregulated TSH receptor expression in human orbital preadipocytes (37). This combined anti-adipogenic and profibrotic response suggests a shift in fibroblast functional behavior that may contribute to progressive tissue remodeling and fibrosis in TED.

The observed upregulation of TGFβ following TNFα stimulation is consistent with previous studies showing that TNFα can potentiate TGFβ signaling in different fibroblasts types, thereby promoting ECM deposition and myofibroblast differentiation (38, 39, 40). Our findings therefore support a model in which TNFα acts as an upstream inflammatory switch linking chronic inflammation directly to fibrotic remodeling within the orbit. This mechanistic connection may be particularly relevant for the development of future therapies aimed at preventing irreversible fibrosis in advanced TED.

Furthermore, TNFα elicited a strong proinflammatory response characterized by increased secretion of IL-6 and IL-8. The induction of IL-6 was significantly more pronounced in mOFs from TSHR-immunized mice compared with β-Gal controls. The calculation of a cytokine index (Δlog_2_(IL-6) − Δlog_2_(IL-8)) demonstrated a shift toward an IL-6-dominant response in TSHR-derived fibroblasts, suggesting that TNFα preferentially enhances IL-6-mediated, profibrotic inflammation. Similar patterns have been observed in other fibroblast populations, where TNFα robustly induces IL-6 and promotes fibroblast activation and fibrosis-related signaling (e.g. synoviocytes, hepatic and cardiac fibroblasts, and CAFs). This underscores the broader relevance of a TNFα–IL-6 axis (41, 42, 43, 44, 45).

In contrast, IFNγ alone did not significantly alter IL-6 or IL-8 secretion, suggesting TNFα as a key inflammatory driver in the orbital microenvironment (46, 47). The identification of a disease-specific TNFα–IL-6 signaling axis is interesting because IL-6-directed therapies are already clinically available, raising the possibility that combined TNFα/IL-6 pathway inhibition could represent a future therapeutic strategy in TED. Consistent with previous studies showing excessive ECM and glycosaminoglycan (GAG) accumulation in TED, our data demonstrate that TNFα enhances HA synthesis in orbital fibroblasts. This effect is also described in a study by Campo *et al.* (48), who showed that TNFα markedly upregulates hyaluronan synthase (HAS) isoforms and stimulates HA production in human skin fibroblasts. Their findings support our results, indicating that TNFα acts as a potent enhancer of HA synthesis across fibroblast populations (48). Increased HA deposition and elevated hyaluronan synthase expression have been described in active TED and are linked to tissue expansion and fibrosis (49, 50, 51). In our model, TNFα significantly increased HA concentrations and HAS2 expression, with a stronger response in mOF from TSHR-immunized mice. These findings suggest that autoimmune sensitization amplifies TNFα-driven ECM remodeling pathways, thereby contributing to pathological orbital tissue expansion.

Collectively, our findings suggest that autoimmune priming enhances TNFα-associated fibroblast activation, thereby linking chronic inflammation to profibrotic remodeling in TED. By integrating chemokine expression, cytokine signaling, adipogenic suppression, and ECM remodeling within one experimental framework, our study provides a more comprehensive mechanistic model of TNFα-driven orbital fibroblast activation in TED.

While current therapies, such as IGF-1R inhibition (e.g. teprotumumab), primarily target specific signaling pathways, our findings suggest that TNFα may function as a broader upstream regulator integrating inflammatory, fibrotic, and ECM-related mechanisms. This makes TNFα particularly attractive as a potential therapeutic target because modulation of this pathway could simultaneously affect multiple disease-driving processes rather than a single downstream signaling cascade. In line with this concept, TNFα inhibitors, such as etanercept, adalimumab, and golimumab, have already demonstrated clinical efficacy in several chronic inflammatory and autoimmune diseases, and preliminary studies in TED reported improvement in inflammatory disease activity following anti-TNFα treatment.

In parallel, emerging therapeutic strategies directly targeting the TSHR pathway, including TSHR-blocking antibodies and small-molecule TSHR antagonists, have shown promising preclinical and early clinical results in GD and TED (52, 53, 54). These approaches aim to inhibit receptor activation upstream of both thyroidal and orbital autoimmune responses and may therefore complement existing anti-inflammatory therapies. Targeting TNFα may be especially relevant in early inflammatory disease stages or in patients who show insufficient responses to IGF-1R- or TSHR-directed therapies. In addition, our data support the concept that combinatorial therapeutic approaches targeting IGF-1R-, TSHR-, and TNFα-mediated pathways may provide superior clinical benefit in treatment-resistant TED. However, despite this strong mechanistic rationale, the clinical use of TNFα inhibitors in TED requires further investigation to evaluate their long-term therapeutic efficacy and safety.

## Declaration of interest

The authors declare that there is no conflict of interest that could be perceived as prejudicing the impartiality of the work reported.

## Funding

This work was supported by the Stiftung Universitätsmedizin Essen, University of Duisburg-Essen, and by Deutsche Forschungsgemeinschaft (DFG) grant GO 3465/1-1 to GEG.

## Author contribution statement

AD conceived the study; designed the methodology; performed validation, formal analysis, investigation, and visualization; and wrote the original draft of the manuscript. AG conceived the study; designed the methodology; performed validation, formal analysis, and investigation; and wrote, reviewed, and edited the manuscript. MH, JK, IN, and FH performed formal analysis and investigation. SL and NB acquired resources and wrote, reviewed, and edited the manuscript. SM contributed expertise and resources. JPB contributed expertise and wrote, reviewed, and edited the manuscript. AE acquired resources and funding; supervised the study; and wrote, reviewed, and edited the manuscript. G-EG conceived the study; designed the methodology; acquired resources and funding; supervised the study; administered the project; and wrote, reviewed, and edited the manuscript. All authors contributed to the article and approved the submitted version.
